# Monitoring Bacterial Community of Human Gut Microbiota Reveals an Increase in *Lactobacillus* in Obese Patients and *Methanogens* in Anorexic Patients

**DOI:** 10.1371/journal.pone.0007125

**Published:** 2009-09-23

**Authors:** Fabrice Armougom, Mireille Henry, Bernard Vialettes, Denis Raccah, Didier Raoult

**Affiliations:** 1 URMITE - UMR CNRS 6236, IRD 3R198, Université de la Méditerranée, Faculté de médecine, Marseille, France; 2 Service de Nutrition, Maladies Métaboliques et Endocrinologie, UMR Université méditerranée-INRA U1260, CHU de la Timone, Marseille, France; 3 Nutrition and Diabetology Department, University Hospital Sainte Marguerite, Marseille, France; Columbia University, United States of America

## Abstract

**Background:**

Studies of the bacterial communities of the gut microbiota have revealed a shift in the ratio of *Firmicutes* and *Bacteroidetes* in obese patients. Determining the variations of microbial communities in feces may be beneficial for the identification of specific profiles in patients with abnormal weights. The roles of the archaeon *Methanobrevibacter smithii* and *Lactobacillus* species have not been described in these studies.

**Methods and Findings:**

We developed an efficient and robust real-time PCR tool that includes a plasmid-based internal control and allows for quantification of the bacterial divisions *Bacteroidetes*, *Firmicutes*, and *Lactobacillus* as well as the methanogen *M. smithii*. We applied this technique to the feces of 20 obese subjects, 9 patients with anorexia nervosa, and 20 normal-weight healthy controls. Our results confirmed a reduction in the *Bacteroidetes* community in obese patients (p<0.01). We found a significantly higher *Lactobacillus* species concentration in obese patients than in lean controls (p = 0.0197) or anorexic patients (p = 0.0332). The *M. smithii* concentration was much higher in anorexic patients than in the lean population (p = 0.0171).

**Conclusions:**

*Lactobacillus* species are widely used as growth promoters in the farm industry and are now linked to obesity in humans. The study of the bacterial flora in anorexic patients revealed an increase in *M. smithii*. This increase might represent an adaptive use of nutrients in this population.

## Introduction

Obesity disorders affect many developed countries and result from an imbalance in energy (input *versus* output) with serious heath consequences, including cardiovascular disease, type II diabetes mellitus, and colon cancer [1], [2]. The role of gut microbiota in obesity is a current public heath problem. The gut environment likely contributes to the energy balance disequilibrium because of its involvement in energy intake, conversion, and storage. The impact of the gut microbiota composition on body weight gain or host adiposity has been supported by reports involving mouse models with a genetic tendency for obesity [3]–[5].

Advances in high-throughput sequencing methods have recently allowed for better characterization of the activities and composition of the complex gut microbial community in both healthy patients and those in disease states [6]–[10]. Large-scale clonal Sanger sequencing of the 16S rRNA genes of gut microbiota [7], [11] have contributed to our understanding of their diversity and ecology. These methods have allowed the identification of a vast number of new and uncultivated species [12]. A recent 16Sr RNA sequencing-based investigation showed that the *Firmicutes*/*Bacteroidetes* (F/B) ratio differed in obese and lean human subjects, mainly due to a reduced *Bacteroidetes* proportion in obese subjects. This finding was unexpected, because the *Bacteroidetes* member *Bacteroides thetaiotaomicron* is associated with host adiposity [3]. Using a weight loss program, Ley and colleagues [7] demonstrated that a decrease in the F/B ratio in obese individuals correlated with weight loss over time and suggested that modulating the abundance of specific bacterial communities might be beneficial in the treatment of obesity. The growth promoters such as *Lactobacillus* species could be one of these specific bacterial communities since its impact on food conversion and weight gain has been demonstrated in farm animals [13].

The characterization and the relative abundance of higher taxonomic orders of gut microbiota have been investigated using multiple methods, including metagenomics, microarrays, and sequencing of large clone libraries. Although these methods are powerful in terms of data production, they involve time-consuming, costly, and tedious data analysis.

In the present work, we identified and used the genomic signature of *Lactobacillus*, *Methanobrevibacter smithii*, *Bacteroidetes*, and *Firmicutes* divisions. We rapidly assessed their relative abundance in the microbiota of obese subjects, lean subjects, and patients with anorexia nervosa using a real-time PCR assay. This real-time PCR assay is a practical, low cost clinical method for monitoring the variations of bacterial phyla of the gut. The genus, species, and divisions we focused on may play a role in the disorder of obesity.

## Methods

### Bacterial Sequences and System Assessment

The sequences of archaea domain, *M. smithii* species, bacterial phyla and genus were downloaded from the ribosomal database Project II (RDP-II) using an online hierarchical browser tool. “Isolates” and “uncultured” sequences that were labeled as “good quality” and had a length greater than 1,200 bp were selected. The *Firmicutes*, *Bacteroidetes* and *M. smithii* systems (the nucleotide sequence “forward primer-probe-reverse primer”) were built as a nucleotide pattern. The pattern was used to search against sequences of phyla and genera using the DREG program of the EMBOSS package.

### Statistical Tests

The *Bacteroidetes* copy number values were converted into logarithm values in order to equalize the standard deviations. The normal distribution was checked using the Shapiro-Wilk test. One-way ANOVA (P value for the Bartlett's test) with the Tukey-Kramer post-test was performed on the *Bacteroidetes* values for the anorexic, obese, and lean groups. A Fisher exact test was performed on a contingency table for the *Lactobacillus* copy number.

An unpaired t-test was used for the comparison of *M. smithii* quantification between the anorexic or the obese group and the lean control group. The statistics were performed using GraphPad Prism version 5 software.

### Patient Recruitment

All the study was approved by the local ethics committee “Comité d'éthique de l'IFR 48, Service de Médecine Légale”, (Faculté de Médecine, 27 Bld Jean Moulin, 13005 Marseille, FRANCE) under the accession number N°08-003, January the 21st, 2009. All patients were recruited at University Hospital (CHU) of Marseille. Only verbal consent was necessary from patients for this study. This is relied on the French bioethics decree N° 2007-1220 published in the official journal of the French Republic. Normal weight control subjects were anonymously recruited from individuals working at the laboratory (or belonging to their family) (n = 20; 13 to 68 years-old; BMI = 20.68 kg/m^2^±2.014). No several members of the same family have been included in the study. Patients were enrolled in inpatient and outpatient clinics at the Department of Nutrition (University Hospital of Marseille, France). Obese patients were selected from patients attending the clinic for surgical treatment of excessive body weight (n = 20, 17 to 72 years-old; BMI = 47.09 kg/m^2^±10.66) and had not taken probiotics before the stool samples were collected. Subjects with anorexia nervosa (defined by DSM-4 criteria) were recruited from patients who were recently hospitalized in the department (n = 9, 19 to 36 years-old; BMI = 12.73 kg/m^2^ ±1.602). The body mass index (BMI) values were represented as mean values±SD.

### Fecal DNA Isolation

Approximately 1 g of sample (two spoonfuls) was suspended in 10 ml of saline buffer and filtered into a Fecal Specimen Filtration Vial (Orion Diagnostica–Fumouze-Division Diagnostics, Levallois Perret, France). A total of 250 µl of the filtrate was transferred in a sterile screw-cap Eppendorf tube containing 0.3 g acid-washed glass beads (Sigma, Saint Quentin Fallavier, France). The tube was shaken in a FastPrep instrument BIO 101 (Qbiogene, Strasbourg, France) at level 6.5 (full speed) for 90 s to accomplish mechanical lysis. The supernatant was collected and incubated overnight at 56°C with 180 µl of the T1 Buffer from NucleoSpin^®^ Tissue Mini Kit (Macherey Nagel, Hoerdt, France) and 25 µl of proteinase K (20 mg/ml). After a second cycle of mechanical lysis, the samples were incubated for 15 min at 100°C. Next, the DNA was extracted using the NucleoSpin^®^ Tissue Mini Kit according to the manufacturer's procedure. Finally, DNA was eluted in 100 µl of elution buffer and stored at −20°C until use. An extraction negative control with 250 µl of sterile water was introduced in each series of the DNA extraction.

### Real-Time Quantitative PCR Assays

The targeted genes, probe sequences, primer sequences (Eurogentec, Seraing, Belgium), and amplicon sizes for the two real-time PCR assays used in this study are summarized in Table 1. The MGB probes and primers of the *Bacteroidetes* and the *Firmicutes* divisions were designed on the basis of the genomic DNA barcodes previously described [14]. The probe and primers of the *Lactobacillus* genus used in this study were previously reported [15]. The *M. smithii* probe and primers (Table 1) were designed using Primer3 v0.4.0 software [16]. Real-time assays were performed in the MX3000™ system (Stratagene Europe, Amsterdam, The Netherlands) using the QuantiTect PCR mix (Qiagen, Courtaboeuf, France). Included in the samples were 5 pmol of primers and probes labeled with FAM or VIC. A total of 5 µl of DNA extracted from stools or a bacterial strain was diluted to 1/10, 1/100, or 1/1000. The dilutions were added to a final volume of 25 µl. The real-time PCR programs for *Bacteroïdetes* detection included 95°C for 15 min and 45 cycles (95°C 30 s, 48°C 45 s, 72°C 1 min). For *Firmicutes* and *Lactobacillus* detections, the programs included 95°C for 15 min and 45 cycles (95°C 30 s, 60°C 1 min). The specificity of the real-time PCR was tested against DNA from 108 bacterial strains. The results of the comparisons are reported in Supplementary Table S1. The detection and quantification of *Lactobacillus* were performed as previously reported [15]. The *Bacteroïdetes* and *Firmicutes* samples were quantified using a quantification plasmid which was constructed as previously described [15].

**Table 1:** *Bacteroidetes* and *Firmicutes* Real-Time PCR System.

Target phylum	Target gene	Amplicon length (bp)	Primer and probe sequences
***Lactobacillus***	**Tuf**	90	FP: TACATYCCAACHCCAGAACG
			RP: AAGCAACAGTACCACGACCA
			Probe: AAGCCATTCTTRATGCCAGTTGAA
***Firmicutes***	**16S rRNA**	179	FP: GTCAGCTCGTGTCGTGA
			RP: CCATTGTAKYACGTGTGT
			Probe: GTCAANTCATCATGCC
***Bacteroidetes***	**16S rRNA**	184	FP: AGCAGCCGCGGTAAT
			RP: CTAHGCATTTCACCGCTA
			Probe: GGGTTTAAAGGG
***M. smithii***	**16S rRNA**	123	FP: CCGGGTATCTAATCCGGTTC
			RP: CTCCCAGGGTAGAGGTGAAA
			Probe: CCGTCAGAATCGTTCCAGTCAG

FP indicates the forward primer, and RP indicates the reverse primer.

### Quantification Plasmid

Chimeric fragments were used for the quantification of *Bacteroïdetes*, *Firmicutes*, and *M. smithii*.

agcagccgcggtaatACGGAGGATCCGAGCGTTATCCGGATTTATT*gggtttaaaggg*AGCGTAGGTGGACTGGTAAGTCAGTTGTGAAAGTTTGCGGCTCAACCGTAAAATTGCAGTTGATACTGTCAGTCTTGAGTACAGTAGAGGTGGGCGGAATTCGTGGTgtagcggtgaaatgcttag**gtcagctcgtgtcgtga** **GATGTTGGGTTAAGTCCCGCAACGAGCGCAACCCTTATTGTTAGTTGCCATCATTTAGTTGGGCACTCTAGCGAGACTGCCGGTGACAAACCGGAGGAAGGTGGGGATGAC** ***gtcaaatcatcatgcc*** **CCTTATGACCTGGGCTacacacgtgctacaatgg** ccgggtatctaatccggttcGCGCCCCTAGCTTTCGTCCCTCA*ccgtcagaatcgttccagtcag*ACGCCTTCGCAACAGGCGGTCCTCCCAGGATTACAGAAtttcacctctaccctgggag .

The fragment length is 485 bp. The *Bacteroïdetes* targeted sequence is in regular style, the *Firmicutes* targeted sequence is in bold style, and the *M. smithii* targeted sequence is in underlined style. The primer sequences are in lowercase and are underlined; the probe sequences are in lowercase and are in italic.

### Reproducibility Assay

The fidelity of the DNA extraction and microbial quantifications were assessed. Three samples of feces from one obese patient (Ob10), one anorexic patient (Ano1), and one control patient (lean11) were defrosted. For each trial, about 1 g of fecal sample was suspended in 5 ml of 0.05 M Tris, pH 7.3. A volume of 250 µL of the suspension was used for the DNA extraction as described in the fecal DNA isolation section. This operation was performed five times and generated five independent DNA extracts per sample. Each DNA extract was tested five times by real-time PCR to determine the quantity of *Firmicutes*, *Bacteroidetes*, and *Lactobacillus*.

## Results

### Sensitivity and Specificity of the “Forward primer-probe-reverse primer” Systems

Oligonucleotide primers and probe sequences from *Bacteroidetes*, *Firmicutes*, *Lactobacillus* and *M. smithii* are shown in Table 1. The sensitivity and the specificity of the *Bacteroidetes*, *Firmicutes* and *M. smithii* systems were assessed *in silico* using the ribosomal RDP-II database [17]. The *Bacteroidetes* system is highly sensitive (89.89%), since it has a perfect match with 30,237 out of the 33,639 16S rRNA sequences of the RDP-II *Bacteroidetes* phylum. The *Firmicutes* system is also highly sensitive (88.94%), because it has a perfect match with 83,576 out of 93,969 of 16S rRNA sequences of the RDP-II *Firmicutes* phylum. The *M. smithii* system has a sensitivity of 88.89% (8 matches out of 9 sequences).

**Figure 1:**
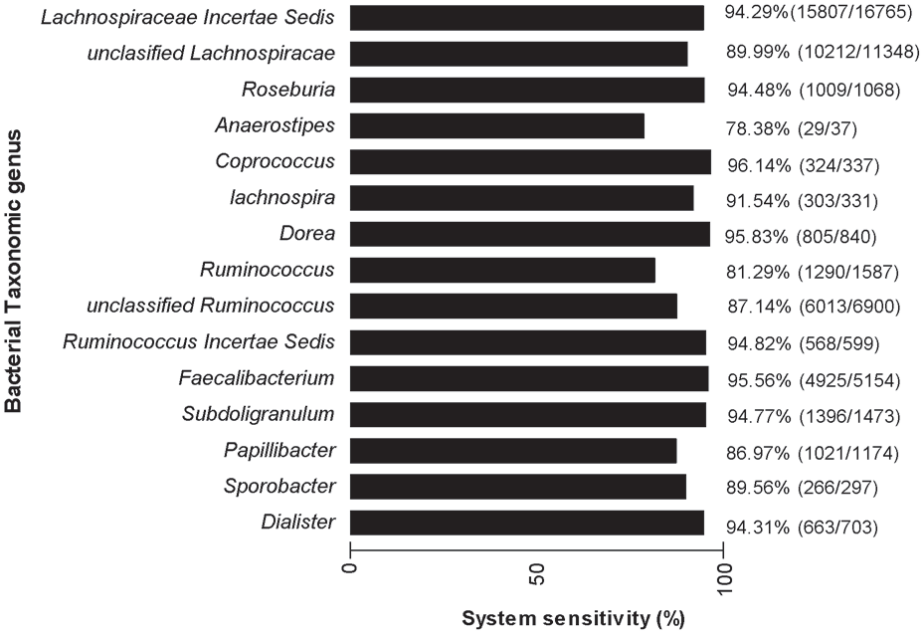
*Firmicutes* system sensitivity at genus taxonomic level. Scores in parentheses indicate the number of sequences matching the system out of the total sequence number of a given genus.

**Figure 2:**
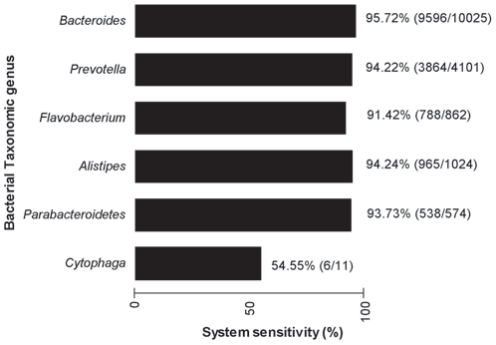
*Bacteroidetes* system sensitivity at genus taxonomic level. Scores in parentheses indicate the number of sequences matching the system out of the total sequence number of a given genus.

The assessment of the system's sensitivity was refined at the genus taxonomic level for *Firmicutes* and *Bacteroidetes*. Although *Anaerostipes* is the least sensitive genus (sequence match % <80) with the *Firmicutes* system (Figure 1), the most abundant genera, including *Lachnospiraceae*, *Faecalibacteriun*, and *Subdoligranulum*, are highly detected (sequence match % >90 for each genus). The 16S rRNA sequences of the *Bacteroidetes* genus were also efficiently recovered by the *Bacteroidetes* system (sequence match % >90), except for the *Cytophaga* genus (Figure 2).

The specificity (or the false discovery rate) of the two systems was evaluated by looking for perfect matches with sequences outside of the *Firmicutes* and *Bacteroidetes* phyla. Scanning all the RDP-II 16S rRNA sequences outside of the *Firmicutes* or the *Bacteroidetes* phyla, the false-discovery rate for the *Firmicutes* and *Bacteroidetes* phyla is 0.83% and 0.01%, respectively. As for the sensitivity, these results suggest a high specificity for the two systems. Finally, the false discovery rate of the *M. smithii* system reaches 0.025% for the archaea domain (2 matches out of 7,873 sequences) and is of 0% for the bacteria domain (no match out of 322,863 sequences).

### Assessment of the Detection of Cultivated Strains from *Bacteroidetes* and *Firmicutes* Phyla

A large collection of strains inhabiting the gut microbiota that were taxonomically assigned to the *Bacteroidetes* and the *Firmicutes* bacterial phyla were tested by real-time PCR (Supplementary Table S1). The *Bacteroidetes* system (Supplementary Table S1, column A) successfully detected all *Bacteroidetes* strains, including *Prevotella*, *Bacteroidetes*, *Parabacteroidetes*, *Captocytophaga*, and *Alistipes*. A few false-positives were identified, including three strains that were assigned to the *Firmicutes* phylum (two *Streptococcus* and one *Enterococcus*). However, the detection of these strains is extremely weak due to the threshold cycle (CT) values, which were close to 40. With regard to the assessment of the *Firmicutes* system (Supplementary Table S1, column B), all the *Firmicutes* strains except *Clostridium perfringens* were identified. In addition, no false-positive results were obtained, suggesting a high specificity for the system. Finally, *Fusobacterium* strains were detected as false-positives by the *Firmicutes* probe system. The detection efficiency of the *Lactobacillus* system has been previously reported using a broad range of *Lactobacillus* strains [15].

### Bacterial Quantification of Different Human Populations

Quantification of the bacterial groups using real-time PCR was performed on three distinct populations, including normal-weight healthy controls (lean), patients exhibiting obesity (BMI≥30 kg/m^2^), and patients suffering from anorexia nervosa (defined by DSM-4 criteria). These populations differed in terms of their body mass indices. The bacterial quantification results for the individuals are showed in Table 2.

**Table 2:** *Bacteroidetes*, *Firmicutes*, *Lactobacillus* and *M. smithii* Quantifications.

Sample	*Bacteroidetes*	*Firmicutes*	*Lactobacillus*	*M. smithii*
Lean1	3.26E+10	4.68E+10	0.00E+00	8.16E+07
Lean2	5.08E+10	4.60E+10	0.00E+00	4.56E+08
Lean3	2.91E+10	5.40E+10	0.00E+00	1.93E+04
Lean4	1.62E+10	1.52E+10	0.00E+00	2.55E+08
Lean5	2.87E+09	9.00E+09	0.00E+00	0.00E+00
Lean6	6.12E+09	1.49E+10	0.00E+00	2.77E+03
Lean7	1.02E+10	1.45E+10	9.64E+05	6.72E+07
Lean8	6.80E+09	1.52E+10	0.00E+00	0.00E+00
Lean9	7.88E+09	1.72E+10	0.00E+00	8.80E+08
Lean10	5.20E+09	3.46E+10	5.44E+07	5.72E+02
Lean11	3.18E+10	2.15E+10	4.80E+04	7.24E+05
Lean12	1.58E+09	2.14E+09	1.08E+04	2.96E+06
Lean13	9.44E+09	1.72E+10	0.00E+00	0.00E+00
Lean14	1.67E+10	4.32E+10	0.00E+00	0.00E+00
Lean15	3.05E+10	3.37E+10	0.00E+00	2.02E+08
Lean16	2.15E+09	1.70E+10	0.00E+00	0.00E+00
Lean17	2.03E+09	4.16E+09	0.00E+00	8.64E+06
Lean18	4.72E+09	1.39E+10	0.00E+00	6.68E+02
Lean19	3.33E+09	1.06E+10	0.00E+00	2.53E+06
Lean20	2.06E+08	4.68E+08	2.37E+04	9.16E+04
**Lean Mean**	1.35E+10	2.16E+10	2.77E+06	9.78E+07
Ob1*	1.93E+10	3.82E+10	4.36E+08	3.62E+06
Ob2	3.85E+09	5.84E+10	3.36E+06	0.00E+00
Ob3*	1.77E+08	3.80E+09	1.29E+06	1.96E+02
Ob4	4.92E+09	9.80E+09	0.00E+00	6.16E+08
Ob5	2.01E+10	2.44E+10	0.00E+00	0.00E+00
Ob6	5.44E+08	4.44E+09	0.00E+00	5.48E+03
Ob7	1.05E+10	1.50E+10	0.00E+00	3.15E+02
Ob8	1.63E+09	6.04E+09	4.28E+07	0.00E+00
Ob9	1.94E+09	7.20E+09	3.61E+08	4.68E+03
Ob10	2.68E+08	1.14E+10	2.21E+07	0.00E+00
Ob11	9.08E+08	8.84E+09	0.00E+00	1.01E+09
Ob12	4.76E+07	2.20E+09	5.28E+06	2.76E+04
Ob13	2.14E+09	1.12E+10	0.00E+00	1.40E+04
Ob14	1.34E+08	1.30E+10	0.00E+00	6.76E+02
Ob15	6.20E+09	2.14E+10	0.00E+00	5.44E+02
Ob16	7.20E+08	2.79E+09	3.96E+06	3.88E+02
Ob17	6.60E+08	6.44E+09	0.00E+00	1.14E+03
Ob18	2.96E+08	5.52E+09	1.96E+05	1.45E+09
Ob19	3.37E+08	6.28E+09	0.00E+00	2.82E+08
Ob20	5.80E+08	1.74E+10	0.00E+00	6.96E+05
Ob Mean	3.76E+09	1.37E+10	4.38E+07	1.68E+08
Ano1	5.60E+08	5.44E+09	5.56E+05	5.08E+03
Ano2	4.72E+08	6.72E+09	0.00E+00	1.17E+04
Ano3	2.84E+09	1.36E+10	2.30E+04	4.48E+08
Ano4	2.44E+10	1.98E+10	0.00E+00	3.82E+08
Ano5	7.12E+09	1.42E+10	0.00E+00	3.19E+03
Ano6	3.59E+10	1.48E+10	0.00E+00	9.40E+08
Ano7	6.24E+09	1.26E+10	0.00E+00	7.12E+02
Ano8	1.09E+10	2.04E+10	0.00E+00	8.84E+08
Ano9	6.60E+09	7.24E+09	1.45E+05	2.08E+09
**Ano Mean**	1.06E+10	1.28E+10	8.04E+04	5.26E+08

Scores are bacteria/archaea copies per g of feces. Ano indicates anorexic individuals and Ob indicates obese individuals. Lean indicates normal weight individuals. The diabetic patients are mentioned by an asterisk (*).

**Figure 3:**
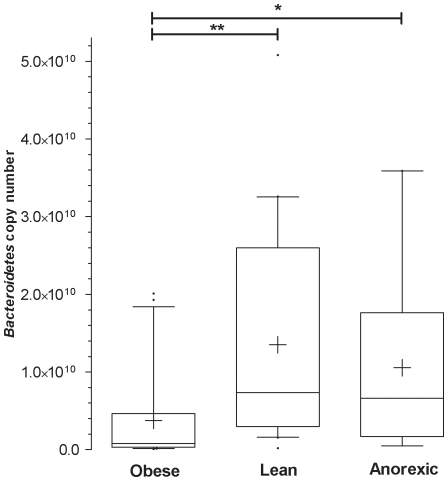
The *Bacteroidetes* quantification. Box and whisker: 10–90 percentiles. Outliers are plotted as a black bubble, means are plotted as a plus, and medians are the black lines in the boxes. P value <0.05 is represented as * and P value <0.01 is represented as ** on the graph.

**Figure 4:**
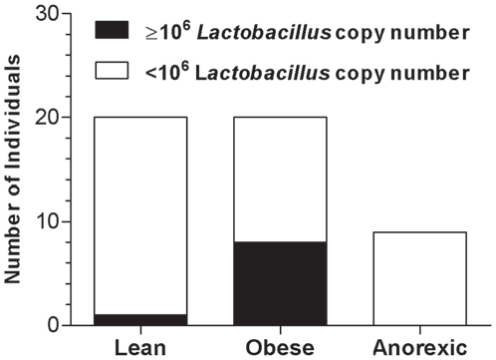
Distribution of high *Lactobacillus* concentrations. The number of individuals having a high *Lactobacillus* concentration was 1 out of 20, 8 out of 20, and 0 out of 9 for the lean, obese, and anorexic groups, respectively.

**Figure 5:**
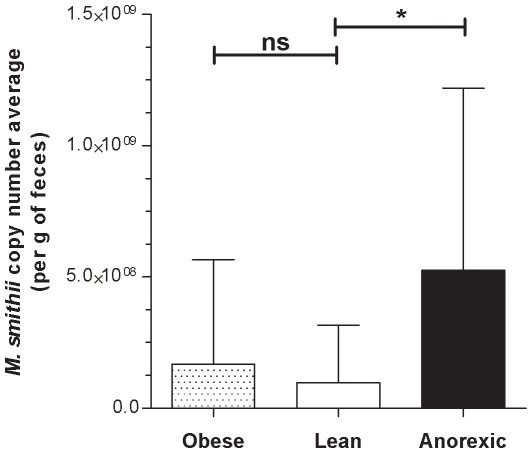
Quantification of the archaeon *M. smithii* species. Values are means±SD. P value <0.05 is represented as * and P value > 0.05 is indicated by “ns” for not significant.

The *Firmicutes* data was similar in the three categories (data not shown). Compared to the lean and anorexic groups, the *Bacteroidetes* data from the obese group had a smaller mean, reduced median and smaller scatter (Figure 3). The differences in *Bacteroidetes* abundance between the obese group and the lean (** p<0.01) or anorexic groups (*p<0.05) were statistically significant (Figure 3). Although the average copy number of *Lactobacillus* was higher in the obese group than the other groups, the difference was not significant. However, at the individual level, the *Lactobacillus* copy number was generally much greater in the obese patients than in the lean or anorexic patients. A contingency bar graph using a threshold of 10^6^ *Lactobacillus* copy numbers confirmed this observation (Figure 4). Based on this threshold, the difference in *Lactobacillus* concentration was significant when obese patients were compared with lean (p = 0.0197) and anorexic (p = 0.0332) patients. The average copy number of the archaeon *M. smithii* is indicated in Figure 5. The *M. smithii* quantification average was slightly higher for the obese group than the lean group as indicated by an obese/lean ratio of 1.72. In addition, the quantification average of *M. smithii* for the anorexic group was much greater than the lean and obese groups by factors of five and three, respectively. Overall, the obese group had a bacterial profile poor in *Bacteroidetes* and rich in *Lactobacillus*. No correlation was found between the quantification of bacterial and archaeal groups and the diabetic state of some obese patients. The anorexic bacterial profile is similar to the lean control group for the *Firmicutes*, *Bacteroidetes*, and *Lactobacillus* but different for *M. smithii* (anorexic group had higher quantification).

## Discussion

The molecular biology method developed in this study aimed to produce a large quantity of data concerning the composition of human gut microbiota. The data was used to analyze bacterial community variations and their correlation with body weight. Using *in silico* primers and probes designed by a pattern identification method [14], our method utilized highly sensitive and specific tests for *Bacteroidetes* and *Firmicutes*. *In silico* and experimental tests demonstrated the efficiency of our real-time PCR assay. To our knowledge, no such benchmarking of the detection quality of the *Firmicutes* and *Bacteroidetes* members has been carried out. The bacterial groups that belong to the *Firmicutes* and *Bacteroidetes* division and the *Lactobacillus* genus were amplified and identified by our method. The system's sensitivity and specificity of the detection of this last bacterial group was previously assessed by Menard et *al.* [15]. The reproducibility of the quantification results obtained from the different samples demonstrates the robustness of the extraction and quantification steps (Table 3). The analysis of five-fold independent DNA extracts indicated low inter-extract variability, with a coefficient of variation ranging from 0.52 to 6.08% for *Bacteroidetes*, from 0.98 to 4.81% for *Firmicutes*, and from 0.64 to 2.67% for *Lactobacillus* (Table 3). In addition, a quantification plasmid allowed for quantification of the bacteria copy number. This method was previously performed in the quantitative evaluation of bacteria genes associated with bioterrorism [18] and vaginosis [15]. The development of our real-time PCR system allowed us to test similar conditions to those described by the Gordon team [4]–[7]. To our knowledge, our study is the largest human study concerning obesity and gut microbiota to date. The 20 obese and 20 lean individuals in this study varied in *Bacteroidetes* number by a magnitude similar to that described by Gordon [4], [7] (Figure 3; p<0.01). This comparison validates our method and reveals that the *Bacteroidetes* profile of French obese patients is similar to that of American obese patients. Nine anorexics patients were also investigated in our study. The results of this group were similar to the lean control group for *Bacteroidetes* (Figure 3), *Firmicutes* (Table 2), and *Lactobacillus* (Table 2 and Figure 4). A major difference was the relative increase in *M. smithii* in anorexic patients compared to the lean control group (Figure 5; p = 0.0171). Accumulation of H_2_ in the human gut reduces the efficiency of microbial fermentation and thereby, the yield of energy. Methanogenic archaea are important since they are involved in the removal of H_2_ excess from the human gut [19]. The most common methanogenic archaeon found in the gut microbiota is *M. smithii* which can reduce CO_2_ with H_2_ to methane. The methanogen identification by cultivation based studies (in human faeces) has been hampered by the strict anaerobic nature of these archaea. The use of culture-independent methods has revealed the high proportion of methanogens [11] that may comprise up to 10% of all anaerobes in the colons of healthy adults [3], [11], [20], [21]. In addition, although methanogens are usually found in 30–50% of Western populations [19], [22], we believe that these proportions are underestimated. Besides, using our method, *M. smithii* was detected in 75% (15 out of 20), 80% (16 out of 20), and 100% (9 out of 9) of the lean, obese, and anorexic faeces, respectively. *Methanobrevibacter* associated with *Bacteroides thetaiotaomicron* led to weight gain in gnotobiotic mice [3]. An increase in *Methanobrevibacter* in obese patients was recently revealed in a study of obese patients before and after gastric bypass by comparing them to lean controls [21]. Our results were in agreement with these previous reports, since our obese group exhibited more *Methanobrevibacter* than our lean group (1.72-fold increase). However, the difference was not significant in our study (p = 0.0883). Surprisingly, the anorexic population showed significantly more *Methanobrevibacter* than lean patients (p = 0.0171). One explanation for this result may be that the development of *Methanobrevibacter* in anorexia nervosa patients might be associated with an adaptive attempt towards optimal exploitation of the very low caloric diet absorbed by these patients. *M. smithii* recycles hydrogen in methane, allowing for an increase in the transformation of nutrients into calories [3]. For anorexic patients, the increase of *Methanobrevibacter* can lead to optimization of food transformation in very low calorie diets. Another explanation for the increase in *Methanobrevibacter* is that it could be related to constipation, which is a common phenomenon in anorexia nervosa patients. An increase in methane producing-bacteria has been demonstrated in patients who suffer from constipation or diverticulosis [23], [24].

**Table 3:** Reproducibility Assay.

Fecal Sample	Bacterial group		Assay extract1	Assay extract2	Assay extract3	Assay extract4	Assay extract5
Obese	*Firmicutes*	mean (Ct)	17.6	17.28	17.12	17.56	18.55
		SD	0.68	0.33	0.32	0.61	0.58
		**CV (%)**	**3.86**	**1.91**	**1.87**	**3.47**	**3.13**
Obese	*Bacteroidetes*	mean (Ct)	16.46	16.22	15.62	16.79	18.35
		SD	0.36	0.78	0.77	0.47	1.35
		**CV (%)**	**2.19**	**4.81**	**4.93**	**2.8**	**7.36**
Obese	*Lactobacillus*	mean (Ct)	24.15	24.49	24.25	24.47	25.08
		SD	0.32	0.42	0.28	0.29	0.67
		**CV (%)**	**1.33**	**1.71**	**1.15**	**1.19**	**2.67**
Anorexic	*Firmicutes*	mean (Ct)	15.84	16.07	16.66	16.37	15.18
		SD	0.43	0.6	0.28	0.16	0.73
		**CV (%)**	**2.71**	**3.73**	**1.68**	**0.98**	**4.81**
Anorexic	*Bacteroidetes*	mean (Ct)	16.34	15.06	16.12	16.27	16.22
		SD	0.64	0.15	0.98	0.73	0.47
		**CV (%)**	**3.92**	**1.00**	**6.08**	**4.49**	**2.9**
Anorexic	*Lactobacillus*	mean (Ct)	26.05	26.14	26.34	26.25	25.14
		SD	0.3	0.22	0.31	0.27	0.16
		**CV (%)**	**1.15**	**0.84**	**1.18**	**1.03**	**0.64**
Lean	*Firmicutes*	mean (Ct)	17.94	17.85	18.12	18.26	17.75
		SD	0.35	0.37	0.38	0.3	0.32
		**CV (%)**	**1.95**	**2.07**	**2.1**	**1.64**	**1.8**
Lean	*Bacteroidetes*	mean (Ct)	15.23	15.33	15.71	14.73	15.27
		SD	0.32	0.22	0.27	0.39	0.08
		**CV (%)**	**2.1**	**1.44**	**1.72**	**2.65**	**0.52**

SD indicates Standard Deviation, means are the Cycle Threshold values (Ct), and CV indicates the Coefficient of Variation. Each assay extract used independent DNA extracts from a given fecal sample. The Ct mean of an assay extract is the result of five replicate real-time PCR measures.

The *Lactobacillus* population inhabiting the gut microbiota was also investigated. In a recent study, we focused on the potential role of *Lactobacillus* in animal weight gain [13]. Recently, child growth was suggested to be associated with *Lactobacillus* intake [25], [26]. Obese populations typically have higher concentrations of *Lactobacillus* than lean (p<0.05) or anorexic (p<0.05) populations (Figure 4). Due to the high inter-subject variability in *Lactobacillus* copy number, only a fraction (8 out of 20) of the obese population had a higher *Lactobacillus* copy number than the lean (1 out of 20) or anorexic (0 out of 9) population. This observation reinforces the possibility of a role for *Lactobacillus* in weight gain. This phenomenon was experimentally confirmed by iterated use of a growth promoter. Recent work by the Gordon team showed that specific enzymatic activities of obese individuals were found in Gram-positive bacteria of the *Firmicutes* phylum (of which *Lactobacillus* belong to) rather than in *Bacteroidetes* [6].

Interestingly, no or few *Lactobacillus* were retrieved from the databank of the analyzed bacteria [7]. The difference in these results can be explained by the difference of bacterial detection sensitivity of the methodologies as well as amplification or cloning bias. Our real time PCR tool is highly sensitive with a bacterial detection threshold of 10^2^ bacteria copies/g of feces. Owing to the relative paucity of the fragments sequenced from studies that use clonal Sanger sequencing of 16S rRNA genes [11], the evaluation of the approximately 10^11^ bacteria copies/g of feces remains superficial by this sequencing method. Therefore, a range of 187 to 1100 fragments sequenced per obese [7] probably does not allow the detection of bacteria in concentrations lower than 10^7^ copies/g of feces (such as the *Lactobacillus*). A recent study found major differences in the gut microbiota of Chinese people compared to American people [27]. Therefore, it is possible that our findings reflect a specific European or French diet.

### Conclusion

This is the first study on the human gut microbiota that shown an increase of *Lactobacillus* in some obese individuals and an increase of *M. smithii* in anorexic patients. Based on these important findings, the use of microarrays for transcriptomic data comparison of *Lactobacillus* and *M. smithii* gene pool between the different populations could be an interesting challenge for the understanding of metabolic activities. However, the purpose of this work was to provide a useful tool for medical monitoring and diagnostic. We developed a molecular biology tool for quantifying the *Bacteroidetes*, *Firmicutes*, *M. smithii*, and *Lactobacillus* microbial groups. This tool allowed for the identification of specific patient microbial communities and the confirmation of a specific profile related to obesity. Patients suffering from anorexia nervosa had a profile similar to that of lean individuals. However, the *M. smithii species* quantification was higher in anorexic patients. Overall, this study provides preliminary data that links *Lactobacillus* levels with obesity.

## Supporting Information

Table S1 Sensitivity and Specificity of the Detection of Firmicutes and Bacteroidetes Strains by Quantitative PCR
(0.14 MB DOC)
