# Supplementary material for: Monitoring Bacterial Community of Human Gut Microbiota Reveals an Increase in Lactobacillus in Obese Patients and Methanogens in Anorexic Patients
Source: PLoS One. 2009 Sep 23;4(9):e7125. doi: 10.1371/journal.pone.0007125 (PMC2742902; doi:10.1371/journal.pone.0007125)
Supplement: Table S1 — Sensitivity and Specificity of the Detection of Firmicutes and Bacteroidetes Strains by Quantitative PCR (0.14 MB DOC) [file pone.0007125.s001.doc]

**Supporting Information**

**Supplementary Table S1. Sensitivity and Specificity of the Detection of *Firmicutes* and *Bacteroidetes* Strains by Quantitative PCR.**

A/ Purified DNA strains analyzed by real-time PCR using *Bacteroïdetes* probe.

B/ Purified DNA strains analyzed by real-time PCR using *Firmicutes* probe.

Results were expressed as cycle threshold (Ct) per PCR.

|  | Strains | A | B |
| --- | --- | --- | --- |
| 1 | *prevotella bivia* | 17.09 | no ct |
| 2 | *prevotella disiens* | 16.7 | no ct |
| 3 | *prevotella oulara* | 16.45 | no ct |
| 4 | *prevotella albensis* | 19.31 | no ct |
| 5 | *prevotella buccae* | 18.64 | no ct |
| 6 | *prevotella denticola* | 16.69 | no ct |
| 7 | *prevotella intermedia* | 17.25 | no ct |
| 8 | *prevotella nigrescens* | 17.99 | no ct |
| 9 | *prevotella melaninogenica* | 16.3 | no ct |
| 10 | *prevotella corporis* | 15.18 | no ct |
| 11 | *prevotella oris* | 36.52 | no ct |
| 12 | *bacteroides fragilis* | 20.37 | no ct |
| 13 | *bacteroides vulgatus* | 20.36 | no ct |
| 14 | *bacteroides thetaiotaomicron* | 21.27 | no ct |
| 15 | *bacteroides ovatus* | 19.35 | no ct |
| 16 | *alistipes finegoldii* | 21.58 | no ct |
| 17 | *alistipes putrenidis* | 20.26 | no ct |
| 18 | *captocytophaga sputigena* | 18.56 | no ct |
| 19 | *captocytophaga ochracea* | 18.16 | no ct |
| 20 | *captocytophaga ochracea* | 18.18 | no ct |
| 21 | *captocytophaga ochracae* | 18.36 | no ct |
| 22 | *captocytophaga haemolytica* | 18.83 | no ct |
| 23 | *captocytophaga granulosa* | 18.62 | no ct |
| 24 | *captocytophaga granulosa* | 20.31 | no ct |
| 25 | *captocytophaga gingivalis* | 20.01 | no ct |
| 26 | *captocytophaga haemolytica* | 22.57 | no ct |
| 27 | *captocytophaga cynodegmi* | 17.15 | no ct |
| 28 | *captocytophaga canimorsus* | 23.04 | no ct |
| 29 | *fusobacterium nucleatum* | 38.6 | 18.36 |
| 30 | *fusobacterium nucleatum* | no ct | 21.86 |
| 31 | *fusobacterium nucleatum* | no ct | 22.15 |
| 32 | *fusobacterium naviforme* | 39.55 | 22.5 |
| 33 | *fusobacterium nucleatum* | no ct | 19.31 |
| 34 | *fusobacterium nucleatum/naviforme* | no ct | 18.54 |
| 35 | *fusobacterium necrophorum* | 38.15 | 22.61 |
| 36 | *Bacillus cereus* | no ct | 18.87 |
| 37 | *Paenibacillus massiliensis* | no ct | 19.1 |
| 38 | *Paenibacillus timonensis* | no ct | 20.73 |
| 39 | *Paenibacillus sanguinis* | no ct | 19.73 |
| 40 | *Streptococcus gordonii* | no ct | 19.49 |
| 41 | *Streptococcus suis* | no ct | 20.2 |
| 42 | *Streptococcus vestibularis* | no ct | 25.39 |
| 43 | *Streptococcus pyogenes* | no ct | 24.84 |
| 44 | *Streptococcus peronis* | no ct | 23.2 |
| 45 | *Streptococcus infantis* | no ct | 19.85 |
| 46 | *Streptococcus cristatus* | no ct | 23.28 |
| 47 | *Streptococcus thermophilus* | no ct | 21.02 |
| 48 | *Streptococcus parasanguinis* | no ct | 19.43 |
| 49 | *Abiotrophia defectiva* | no ct | 19.62 |
| 50 | *Gemella sanguinis* | no ct | 18.9 |
| 51 | *Gemella bergeri* | no ct | 20.48 |
| 52 | *Enterococcus avium* | no ct | 19.73 |
| 53 | *Enterococcus gilvus* | no ct | 22.2 |
| 54 | *Enterococcus hirae* | no ct | 21.56 |
| 55 | *Enterococcus faecalis* | no ct | 22.16 |
| 56 | *Enterococcus raffinosus* | 36.51 | 28.54 |
| 57 | *Enterococcus casseliflavus* | no ct | 23.38 |
| 58 | *Enterococcus durans* | no ct | 20.81 |
| 59 | *Staphylococcus aureus* | no ct | 25.59 |
| 60 | *Corynebacterium accolens* | no ct | 41.35 |
| 61 | *Corynebacterium afermentans sub afermentans* | no ct | 39.7 |
| 62 | *Corynebacterium afermentans sub lipophilum* | no ct | 39.99 |
| 63 | *Corynebacterium amycolatum* | no ct | 39.51 |
| 64 | *Corynebacterium coyleae* | no ct | 39.6 |
| 65 | *Corynebacterium seminale* | no ct | 32.28 |
| 66 | *Corynebacterium urealyticum* | 39.73 | 43.96 |
| 67 | *Staphylococcus capitis* | no ct | 22.53 |
| 68 | *Staphylococcus arlettae* | no ct | 21.47 |
| 69 | *Staphylococcus hominis* | no ct | 23.81 |
| 70 | *Staphylococcus simulans* | no ct | 21.61 |
| 71 | *Staphylococcus caprae* | no ct | 24.89 |
| 72 | *Streptococcus mutans n°162000* | 40.59 | 27.67 |
| 73 | *Streptococcus oralis n°162001* | no ct | 20.7 |
| 74 | *Streptococcus salivarius n°162004* | 38.27 | 28.67 |
| 75 | *Streptococcus sanguinis n°162005* | no ct | 26.3 |
| 76 | *Streptococcus constellatus n°162020* | no ct | 29.48 |
| 77 | *Streptococcus mitis n°162026* | no ct | 26.03 |
| 78 | *Lactobacillus graminis CIP105164* | no ct | 29.99 |
| 79 | *Lactobacillus acidophilus CIP7613* | no ct | 28.9 |
| 80 | *Lactobacillus crispatus CIP 102990* | no ct | 24.51 |
| 81 | *Lactobacillus equi CIP107382* | no ct | 25.49 |
| 82 | *Lactobacillus iners CIP105923 lot 706* | no ct | 27 |
| 83 | *Enterococcus faecium CIP 103510 lot 0203* | no ct | 27.5 |
| 84 | *Enterococcus faecium CIP104104 lot 1102* | no ct | 26.27 |
| 85 | *Enterococcus faecalis CIP103015* | no ct | 17.23 |
| 86 | *Bacillus odysseri CIP 108263 lot18204* | no ct | 23.8 |
| 87 | *Bacillus neidei CIP 107432 lot1082* | no ct | 22.13 |
| 88 | *Bacillus pycnus CIP107434lot1089* | no ct | 24.29 |
| 89 | *Clostridium perfringens* | no ct | no ct |
| 90 | *Clostridium innocuum* | no ct | 20.32 |
| 91 | *Clostridium paraputrificum* | no ct | 15.72 |
| 92 | *Bacteroides uniformis* | 15.47 | no ct |
| 93 | *Bacteroides vulgatus* | 16.52 | no ct |
| 94 | *Bacteroides caccae* | 15.17 | no ct |
| 95 | *Clostridium beijerinckii* | no ct | 18.63 |
| 96 | *Clostridium putrificum* | no ct | 16.69 |
| 97 | *Clostridium septicum* | no ct | 19.89 |
| 98 | *Clostridium perfringens* | no ct | 15.8 |
| 99 | *Clostridium histolyticum* | no ct | 16.33 |
| 100 | *Clostridium difficile* | no ct | 16.91 |
| 101 | *Clostridium bifermentans* | no ct | 22.04 |
| 102 | *Clostridium thiosulforeducens* | no ct | 14.21 |
| 103 | *Clostridium saccharolyticum* | no ct | 15.6 |
| 104 | *Clostridium butyricum* | no ct | 12.89 |
| 105 | *Acidaminococcus fermentans* | no ct | 20.15 |
| 106 | *Acidaminococcus intestini* | no ct | 25.65 |
| 107 | *Parabacteroides distasonis* | 9.62 | no ct |
| 108 | *Peptostreptococcus anaerobius* | no ct | 16.54 |
| 109 | *Eubacterium saburreum* | no ct | 24.06 |
